# Prediction of Severe Disease in Children with Diarrhea in a Resource-Limited Setting

**DOI:** 10.1371/journal.pone.0082386

**Published:** 2013-12-03

**Authors:** Adam C. Levine, Richard M. Munyaneza, Justin Glavis-Bloom, Vanessa Redditt, Hannah C. Cockrell, Bantu Kalimba, Valentin Kabemba, Juvenal Musavuli, Mathias Gakwerere, Jean Paul de Charles Umurungi, Sachita P. Shah, Peter C. Drobac

**Affiliations:** 1 Warren Alpert Medical School, Brown University, Providence, Rhode Island, United States of America; 2 Department of Community Health, Rwanda Ministry of Health, Kigali, Kigali Province, Rwanda; 3 Department of Family and Community Medicine, University of Toronto, Toronto, Ontario, Canada; 4 Watson Institute for International Studies, Brown University, Providence, Rhode Island, United States of America; 5 Department of Medicine, Kirehe Hospital, Kirehe, Eastern Province, Rwanda; 6 Department of Medicine, Butaro Hospital, Butaro, Northern Province, Rwanda; 7 Department of Public Health, University of New South Wales, Randwick, New South Wales, Australia; 8 Division of Emergency Medicine, University of Washington Medical Center, Seattle, Washington, United States of America; 9 Division of Global Health Equity, Brigham and Women’s Hospital, Boston, Massachusetts, United States of America; 10 Research Department, Partners in Health/Inshuti Mu Buzima, Rwinkwavu, Eastern Province, Rwanda; University of Alabama at Birmingham, United States of America

## Abstract

**Objective:**

To investigate the accuracy of three clinical scales for predicting severe disease (severe dehydration or death) in children with diarrhea in a resource-limited setting.

**Methods:**

Participants included 178 children admitted to three Rwandan hospitals with diarrhea. A local physician or nurse assessed each child on arrival using the World Health Organization (WHO) severe dehydration scale and the Centers for Disease Control (CDC) scale. Children were weighed on arrival and daily until they achieved a stable weight, with a 10% increase between admission weight and stable weight considered severe dehydration. The Clinical Dehydration Scale was then constructed post-hoc using the data collected for the other two scales. Receiver Operator Characteristic (ROC) curves were constructed for each scale compared to the composite outcome of severe dehydration or death.

**Results:**

The WHO severe dehydration scale, CDC scale, and Clinical Dehydration Scale had areas under the ROC curves (AUCs) of 0.72 (95% CI 0.60, 0.85), 0.73 (95% CI 0.62, 0.84), and 0.80 (95% CI 0.71, 0.89), respectively, in the full cohort. Only the Clinical Dehydration Scale was a significant predictor of severe disease when used in infants, with an AUC of 0.77 (95% CI 0.61, 0.93), and when used by nurses, with an AUC of 0.78 (95% CI 0.63, 0.93).

**Conclusions:**

While all three scales were moderate predictors of severe disease in children with diarrhea, scale accuracy varied based on provider training and age of the child. Future research should focus on developing or validating clinical tools that can be used accurately by nurses and other less-skilled providers to assess all children with diarrhea in resource-limited settings.

## Introduction

There were 1.7 billion cases of diarrhea in children in 2011, resulting in 36 million cases of severe disease and 700,000 deaths, or more than 10% of all child deaths worldwide[1]. As the severity of diarrheal disease in children varies widely, accurately assessing dehydration status remains a crucial step in preventing mortality[2]. While children with severe dehydration require immediate treatment with intravenous fluids to prevent hemodynamic compromise, organ ischemia, and death, children with mild to moderate dehydration have a significant reduction in hospital length of stay and fewer adverse events when treated with oral rehydration solution (ORS) alone[3]. For children with mild to moderate dehydration, ORS can also be more cost-effective than intravenous fluids, especially in resource-limited settings.

Overall, only 2% of diarrhea cases in children will progress to severe disease (defined as severe dehydration or death)[1]. However, there are few tools available to help providers in resource-limited settings predict which children with diarrhea are at risk for severe disease and require hospital admission and intravenous fluids. Prior research has found that no single laboratory test or clinical sign has demonstrated adequate sensitivity, specificity, and reliability for detecting severe dehydration in children[4]. To overcome the limited accuracy of individual clinical signs, the World Health Organization (WHO) recommends using a combination of four different clinical signs to identify severe dehydration in children with diarrhea, which is considered the standard of care in many low-income countries[5]. Alternatively, the United States Centers for Disease Control (CDC) recommend that providers use a more complex scale of 12 signs and symptoms for evaluating dehydration status in children with diarrhea[6]. 

Several studies conducted in urban referral hospitals in high- and middle-income countries have found that clinical scales composed of various combinations of signs and symptoms may be accurate predictors of dehydration in children with diarrhea, including the Clinical Dehydration Scale, originally developed in Canada[7-10]. However, the accuracy of these clinical scales have not been previously validated against an accepted criterion standard in a low-income country setting, where the vast majority of diarrhea morbidity and mortality occurs worldwide, and where differences in provider training and diarrhea etiology may compromise the accuracy of clinical scales derived in wealthier countries.

In this study, we investigate the accuracy of three clinical scales, including the WHO severe dehydration scale[5], the CDC scale[6], and the Clinical Dehydration Scale[9] for predicting severe disease in children with diarrhea admitted to three government hospitals in Rwanda, a low-income country in sub-Saharan Africa. We also perform sub-group analyses to determine if the accuracy of these clinical scales varies based on the type of provider performing the exam and the age of the child being examined.

## Methods

### Study Design

The data presented in this manuscript was collected as part of the Clinical Exam and Ultrasound for Dehydration Evaluation (CUDE) study, which prospectively enrolled a non-consecutive cohort of children admitted with diarrhea to three rural hospitals in Rwanda. The specific aims of the CUDE study were to test the accuracy of the WHO severe dehydration scale, the CDC scale, and ultrasound of the inferior vena cava for detecting severe disease in children with diarrhea in a resource-limited setting. In this paper, we present data on the accuracy of the clinical prediction rules; data on ultrasound performance will be reported separately. 

### Ethics Statement

This study was approved by the Rwanda National Ethics Committee and the Lifespan (Rhode Island Hospital) Institutional Review Board and conducted in accordance with the STARD guidelines for diagnostic studies[11]. Written informed consent was obtained from the parent or guardian of all children enrolled in the study, except in cases where the parent or guardian could not read or write. Both the Rwanda National Ethics Committee and Lifespan Institutional Review Board allowed for verbal consent in these cases, which was documented by the parent or guardian marking the consent form with an X.

### Study Setting

Enrollment for this study took place from December 2010 to April 2012 at three district (first-level) government hospitals in Rwanda. These hospitals are operated by the Rwanda Ministry of Health and all serve rural and relatively impoverished populations, with a combined catchment area of approximately 866,000 people[12].

### Staff Training

All physicians at our study hospitals were general practitioners who had completed five years of medical school and a one-year internship. All nurses had completed either a two or three year nursing diploma or certificate program. All physicians and nurses working at our study hospitals had received training during medical school and nursing school in the assessment and management of children with diarrhea. Prior to study inception we conducted one-hour didactic trainings for all nurses and physicians at each study hospital describing the study goals and procedures, as well as reviewing Rwanda Ministry of Health (MOH) guidelines for managing diarrhea in children, which are based on WHO treatment protocols[13]. We did not attempt to provide additional, in-depth training for hospital staff in the clinical assessment of dehydration in children, as we specifically wanted to investigate how clinical scales performed in a real-world setting. 

### Selection of Participants

All children admitted to the pediatric wards of our study hospitals with acute diarrhea (defined as three or more loose stools per day for one to fourteen days, as per WHO guidelines) were eligible for inclusion, regardless of their final discharge diagnosis[13]. We chose to use the WHO definition of acute diarrhea as our inclusion criterion, as opposed to a specific diagnosis such as gastroenteritis, since it could be easily assessed by study staff on patient arrival to the hospital. Only admitted patients were enrolled in the study, since our criterion standard for severe dehydration required obtaining the daily weight of enrolled patients until they were fully rehydrated. Patients were only enrolled on weekdays from 7:00AM to 5:00PM, based on the availability of study staff, who screened all children arriving at the hospital during study hours. Upon identifying an eligible child, either written or verbal consent was obtained from the parent or guardian of the child in the local language, Kinyarwanda, depending on their literacy level.

### Methods and Measurement

After enrollment, study staff weighed each child, without clothing, using a standard, calibrated study scale (Salter/Breck 235-6S) and obtained basic demographic information from their parent or guardian. Study staff then identified an available hospital physician or nurse to record the signs and symptoms of dehydration that compose the WHO severe dehydration scale and CDC scale (Table 1), followed by their overall clinical impression as to whether the child had severe dehydration. Each patient was assessed clinically by a single provider, either a nurse or physician, based solely on which type of provider happened to be most available at the time of patient enrollment in the study. Study procedures were not allowed to delay immediately necessary interventions, such as delivery of intravenous fluids. Enrollment data was generally collected within one hour of patient arrival to the hospital; in cases where intravenous hydration was begun soon after patient arrival to the hospital, enrollment data was collected immediately after the initiation of intravenous hydration. 

**Table 1:** Clinical scale characteristics and points.

**Characteristic**	**0 Points**	**1 Point**	**2 Points**
**WHO Severe Scale^*a*^**			
**Mental status**	Alert or restless	Lethargic or unconscious	
**Eyes**	Normal	Sunken	
**Thirst**	Drinks normally or thirsty/eager to drink	Not able to drink or drinking poorly	
**Skin pinch**	Skin pinch goes back quickly or slowly	Skin pinch goes back very slowly	
**CDC Scale^*b*^**			
**Mental status**	Well and alert	Restless or irritable	Lethargic or unconscious
**Thirst**	Drinks normally	Thirsty/eager to drink	Unable to drink
**Heart rate**	Normal	Increased	Tachycardia
**Quality of pulses**	Normal	Decreased	Weak or impalpable
**Breathing**	Normal	Fast	Deep
**Eyes**	Normal	Slightly sunken	Very sunken
**Tears**	Tears present	Decreased tears	Absent tears
**Mouth and tongue**	Moist	Dry	Very dry
**Skin fold**	Instant recoil	Recoil in < 2 seconds	Recoil in > 2 seconds
**Capillary refill**	Normal	Prolonged	Minimal
**Extremities**	Warm	Cool	Cold, mottled, or cyanotic
**Urine output**	Normal	Decreased	Minimal
**Clinical Dehydration Scale^*c*^**			
**General appearance**	Normal	Thirsty, restless, or irritable	Lethargic or unconscious
**Eyes**	Normal	Slightly sunken	Very sunken
**Mucous membranes**	Moist	Dry	Very dry
**Tears**	Tears present	Decreased tears	Absent tears

*a* Adapted from: World Health Organization. The treatment of diarrhoea: a manual for physicians and other senior health workers. 4th rev. 2005. Severe dehydration defined as two or more points.

*b* Adapted from: King CK, Glass R, Bresee JS, Duggan C. Managing Acute Gastroenteritis Among Children. MMWR. 2003;52(RR16):1-16.

*c* Adapted from: Parkin PC, Macarthur C, Khambalia A, Goldman RD, Friedman JN. Clinical and laboratory assessment of dehydration severity in children with acute gastroenteritis. Clin Pediatr (Phila). 2010 Mar;49(3):235–9.

**Figure 1:**
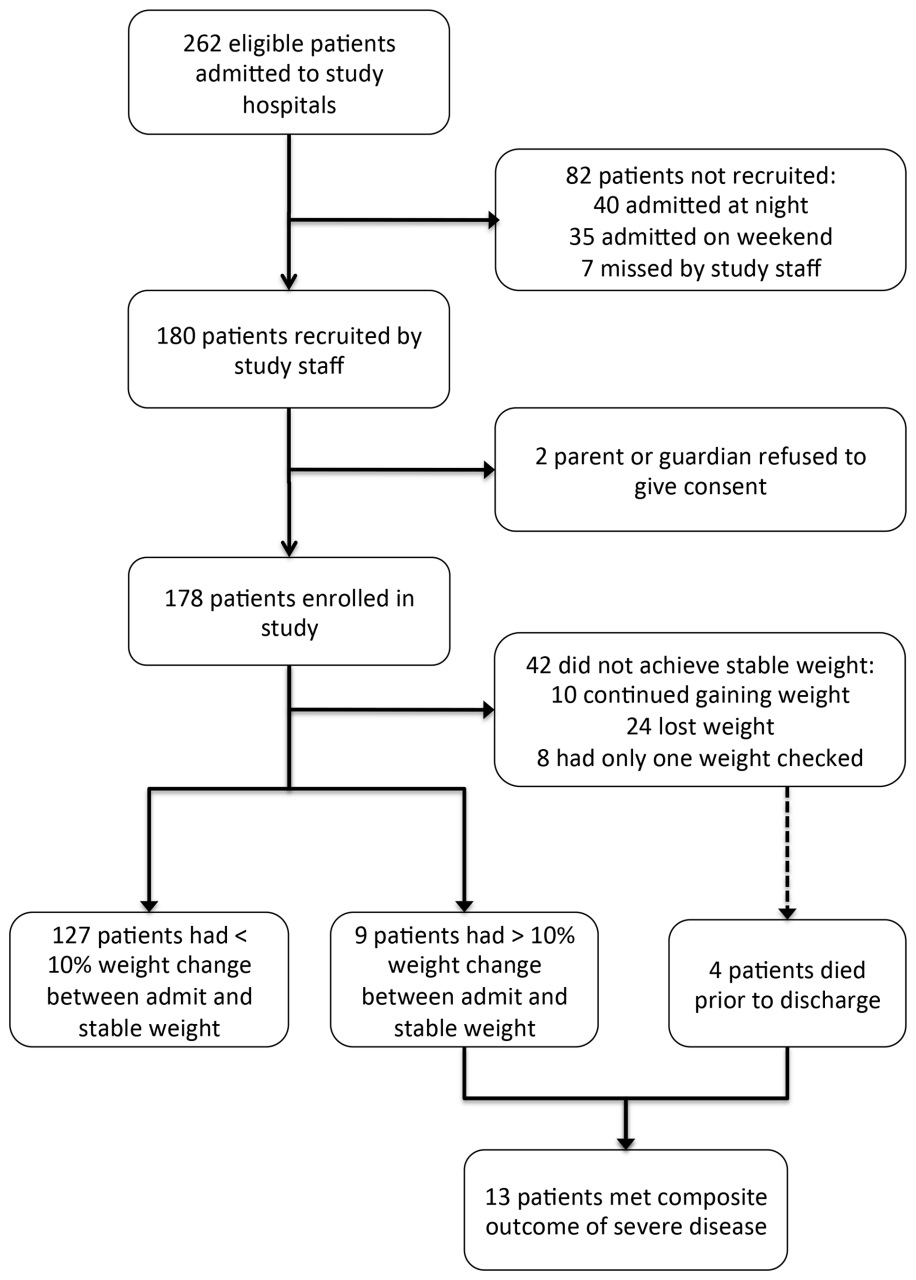
Patient enrollment at study hospitals.

After enrollment and initial management, the patient was admitted to the hospital and rehydrated according to standard Rwanda MOH protocols. All treatments, including oral rehydration, intravenous fluids, and antibiotics, were administered at the discretion of the treating provider. Study staff followed all children during their hospitalizations, repeating weight measurements daily using the same study scale, without clothing, at 7:00am each morning. Study staff also checked the pediatric ward each day for otherwise eligible patients who had not been enrolled on arrival, in order to determine the percentage of eligible patients enrolled (Figure 1). Upon discharge, study staff collected data from the patient’s hospital chart on final discharge diagnosis and clinical assessment of severe malnutrition. 

### Outcomes

**Figure 2:**
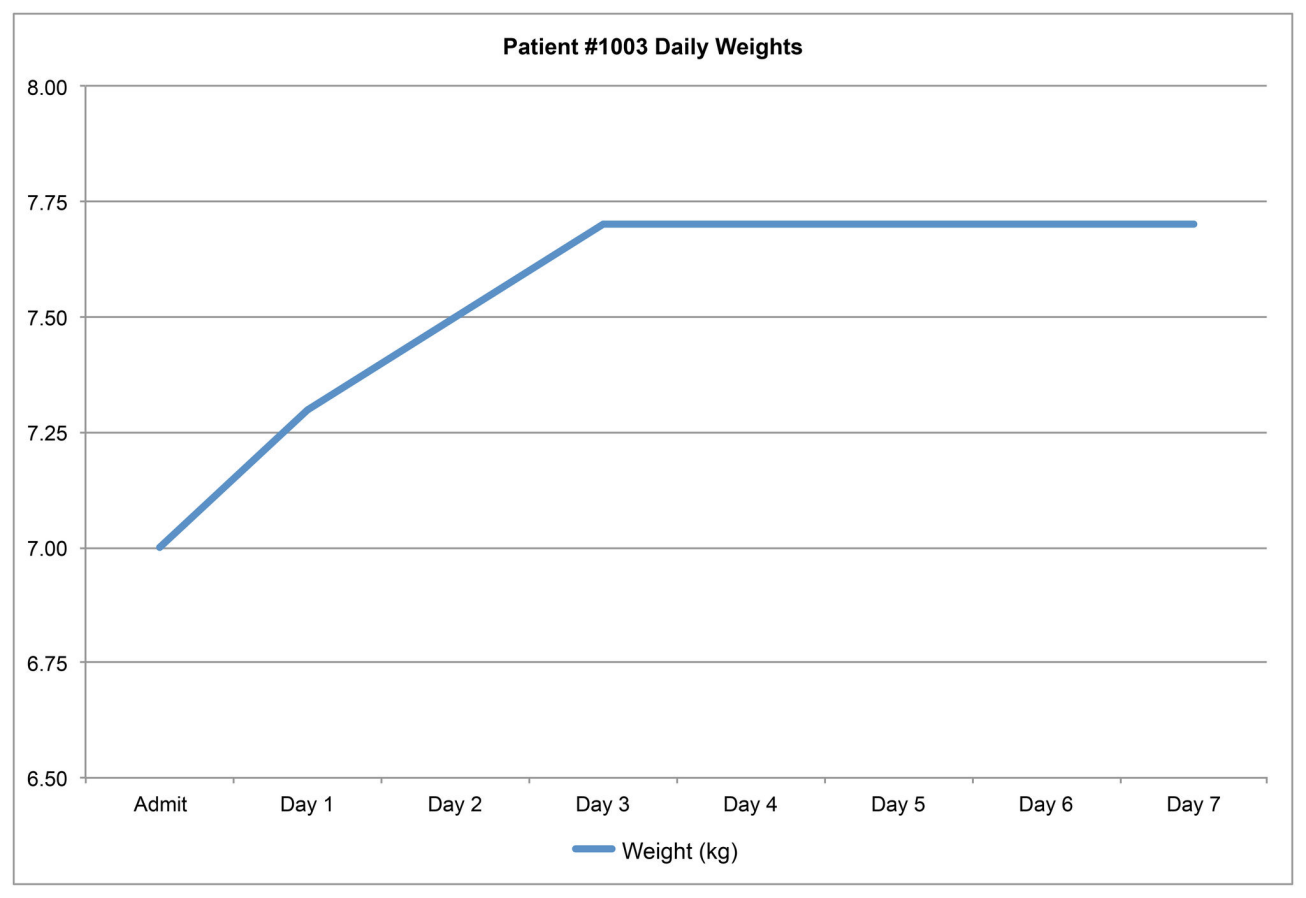
Daily weights for sample study patient.

For each patient enrolled, we determined the highest two consecutive weight measurements that differed by less than 2%, with the mean of these weights used as the stable weight based on standards in the literature[4,8]. Figure 2 shows the daily weights for the first patient in our study to achieve a stable weight, demonstrating how children with dehydration will rapidly gain weight as they are rehydrated until they achieve their pre-illness weight, at which point they will stop gaining weight as their kidneys diurese excess fluid. We were not able to determine stable weights for children who continued gaining weight until discharge, children who lost weight during their admission, and children who had only a single weight measured. For each child who achieved a stable weight, we calculated the percent weight change with rehydration, our proxy for percent volume lost due to diarrhea, using the following equation: (Stable Weight – Admission Weight) / Stable Weight * 100%. A percent weight change of 10% or more was considered to be severe dehydration based on WHO guidelines[13]. 

Our primary outcome of interest was severe disease, defined in the global child health literature as either severe dehydration or death[1]. Based on prior data collected in our setting, we expected a 2% case fatality ratio for patients enrolled in the study[14,15]. However, these children would not meet our criterion standard for severe dehydration, as they were unlikely to survive long enough to be rehydrated to a stable weight. Since removing these children from analysis would create bias by excluding the most severely dehydrated children, we included both children who met our criterion standard for severe dehydration and children who died in our composite outcome of severe disease.

We used the four clinical signs recommended by WHO to construct the WHO severe dehydration scale (Table 1)[5]. We used the twelve signs recommended by the CDC for construction of the CDC scale[6]. Because the CDC scale is a qualitative scale, we created point assignments for each sign or symptom in order to be able to measure its accuracy against our criterion standard of severe disease, assigning 0 points to signs in column 1, 1 point to signs in column 2, and 2 points to signs in column 3 (Table 1). Although we did not prospectively collect data to assess the Clinical Dehydration Scale, we were able to construct this 8-point scale as recommended by Parkin, et al. after the conclusion of the study using the clinical signs collected for the WHO severe dehydration scale and CDC scale (Table 1)[9]. 

### Analysis

We constructed receiver-operating characteristic (ROC) curves to evaluate the performance of the WHO severe dehydration scale, the CDC scale, and the Clinical Dehydration Scale, compared to our composite outcome of severe disease. In addition, we calculated the test characteristics, including sensitivity, specificity, positive likelihood ratio (LR+) and negative likelihood ratio (LR-), for each scale using the best cut-points for severe disease obtained from our ROC curve analysis. In determining the best cut-points for each clinical scale, we attempted to maximize sensitivity, given the importance of not missing children with severe disease, while still maintaining specificity above 50%.

We conducted exploratory subgroup analyses to investigate whether the performance of the three clinical scales differed based on the age of the child (younger than or older than twelve months) and the type of provider who performed the physical exam (physician or nurse). Finally, we evaluated the test characteristics of overall clinical impression for predicting severe disease. 

Statistical analyses were performed using SPSS version 20.0 (IBM, Armonk, NY). Comparisons of baseline characteristics in sub-groups were conducted using the Mann-Whitney U test for continuous, non-parametric data and the Chi Square test for categorical data. Standard algorithms from the statistical literature were used to calculate likelihood ratios and their confidence intervals[16]. 

### Sample Size

We used the algorithms developed by Hanley, et al. for comparing the areas under ROC curves (AUCs) to estimate our sample size with a Type I error of 0.05 and a power of 0.80[17]. Based on a difference of about 0.2 in the AUCs of the best and worst ROC curves in our pilot study, we powered our current study to detect a difference of at least 0.2 in the AUCs of our different scales. Using the Hanley algorithms and the proportion of children with severe dehydration or death in our pilot study, we calculated a minimum required sample of 160 children with diarrhea[14,15]. 

## Results

### Characteristics of Study Subjects

Overall, 178 children were enrolled in our study; the most common reason that otherwise eligible children were not enrolled was arrival at night or on weekends (Figure 1). The median age for enrolled children was 10 months (range 1 month – 12 years); 96% were under 5 years and 92% were under 3 years of age. Twenty-six patients (15%) had clinical signs of severe malnutrition (either severe wasting or edema recorded in their hospital chart) and 109 children (62%) were discharged with a primary diagnosis of gastroenteritis. Four children (2.3%) died during their hospitalization.

Of the 178 children enrolled, 136 (76%) achieved a stable weight prior to discharge, allowing for calculation of their percent weight change with rehydration. Ten (6%) continued gaining weight until discharge, while 24 (13%) lost weight during their hospitalization, and 8 (5%) had only one weight measured as they were admitted for less than 24 hours. Children required a median of 2 days to achieve a stable weight, with three quarters of children achieving a stable weight within 3 days of admission. Children who did not achieve a stable weight prior to discharge were similar to those who did in terms of age, gender, and final discharge diagnosis, though they were significantly more likely to be malnourished (Table 2).

**Table 2:** Comparison of patient baseline characteristic by stable weight category.

**Characteristic**	**Achieved Stable Weight (n=136)**	**Did Not Achieve Stable Weight (n=42)**	**Significance (p-value)**
**Age, median, months**	9.5	12	0.130
**Male sex (%)**	59	60	0.936
**Gastroenteritis (%)**	64	55	0.304
**Severe Malnutrition (%)**	10	29	0.003

Of the 136 children who achieved a stable weight prior to discharge, the average percent weight change with rehydration was 3.2%. Of these children, 70 (51%) had no dehydration (less than 2% weight change), 57 (42%) had some dehydration (2-10% weight change), and 9 (7%) had severe dehydration (greater than 10% weight change). Therefore, a total of 13 children met our composite outcome of either severe dehydration (9) or death (4), as described in Figure 1.

### Accuracy of Clinical Scales

**Figure 3:**
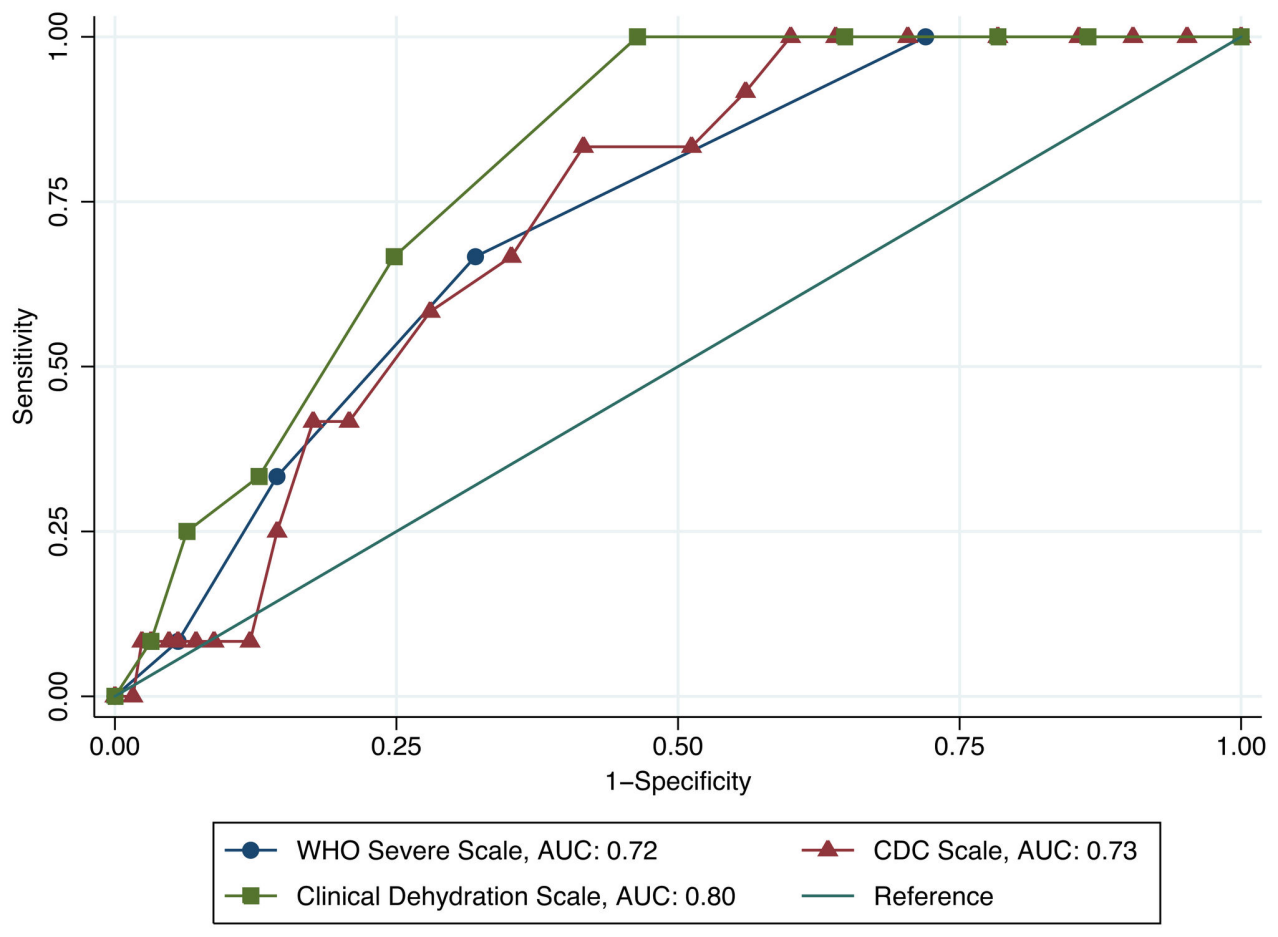
ROC curves for clinical scales as predictors of severe disease.

Figure 3 demonstrates the ROC curves for each of the three clinical scales. The WHO severe dehydration scale, CDC scale, and Clinical Dehydration Scale were all moderate predictors of our composite outcome of severe disease in this cohort of children, with AUCs of 0.72 (95% CI 0.60, 0.85), 0.73 (95% CI 0.62, 0.84), and 0.80 (95% CI 0.71, 0.89), respectively. Table 3 summarizes the test characteristics, including sensitivity, specificity, LR+, and LR- for each of the three scales using the best cut-points obtained from our ROC curve analysis. 

**Table 3:** Test characteristics of clinical scales for predicting severe disease using best cut-points.

**Clinical scale (and cut-point)**	**Sensitivity (%)**	**Specificity (%)**	**Positive Likelihood Ratio**	**Negative Likelihood Ratio**
**WHO Severe Scale: ≥2 points (95% confidence interval)**	67 (40-93)	68 (60-76)	2.1 (1.3-3.4)	0.49 (0.2-1.1)
**CDC Scale: ≥10 points (95% confidence interval)**	83 (62-100)	58 (50-67)	2.0 (1.4-2.8)	0.29 (0.1-1.0)
**Clinical Dehydration Scale: ≥4 points (95% confidence interval)**	100 (74-100)	54 (45-62)	2.2 (1.8-2.6)	0.0 (NC)

### Subgroup Analysis by Age of Child

Limiting our analysis to the age ranges for which the individual scales were originally intended—under five years for the WHO severe dehydration scale (AUC 0.70) and under three years for the Clinical Dehydration Scale (AUC 0.79)—did not affect the performance of the two scales as predictors of severe disease. Limiting our analysis to infants less than twelve months, however, did affect the performance of the clinical scales. While all three scales were statistically significant predictors of severe disease in older children, only the Clinical Dehydration Scale remained a statistically significant predictor of severe disease in infants (Table 4).

**Table 4:** Accuracy of clinical scales for predicting severe disease by provider type and child age.

**Clinical scale**	**Area under ROC curve**	**95% confidence interval lower bound**	**95% confidence interval upper bound**
**Full Cohort**			
**WHO Severe Scale**	0.722	0.598	0.846
**CDC Scale**	0.726	0.616	0.836
**Clinical Dehydration Scale**	0.801	0.710	0.892
**Scale Recorded by Nurse**			
**WHO Severe Scale**	0.651	0.470	0.833
**CDC Scale**	0.607	0.423	0.790
**Clinical Dehydration Scale**	0.778	0.632	0.925
**Scale Recorded by Doctor**			
**WHO Severe Scale**	0.780	0.602	0.959
**CDC Scale**	0.827	0.683	0.972
**Clinical Dehydration Scale**	0.830	0.720	0.940
**Age under 12 months**			
**WHO Severe Scale**	0.646	0.462	0.830
**CDC Scale**	0.632	0.448	0.816
**Clinical Dehydration Scale**	0.772	0.610	0.933
**Age over 12 months**			
**WHO Severe Scale**	0.798	0.646	0.951
**CDC Scale**	0.857	0.750	0.965
**Clinical Dehydration Scale**	0.863	0.760	0.966

### Subgroup Analyses by Clinician Training

Ninety-seven (55%) of enrolled children were evaluated clinically by a physician and 81 (45%) were evaluated by a nurse. There were no significant differences in demographic or clinical characteristics between the children assessed by physicians and those assessed by nurses (Table 5). However, there were differences in the performance of the clinical scales by type of provider. While all three scales were statistically significant predictors of severe disease when used by physicians, only the Clinical Dehydration Scale was a statistically significant predictor of severe disease when used by nurses (Table 4).

**Table 5:** Comparison of patient baseline characteristic by examining provider.

**Characteristic**	**Examined by Nurse (n=81)**	**Examined By Doctor (n=97)**	**Significance (p-value)**
**Age, median, months**	9	12	0.115
**Male sex (%)**	63	56	0.325
**Gastroenteritis (%)**	64	60	0.568
**Severe dehydration (%)**	8	6	0.630
**Death (%)**	2	2	0.872

### Overall Clinical Impression

Overall clinical impression was not a statistically significant predictor of severe disease, with a LR+ of 1.54 (95% CI 0.63, 3.74) and a LR- of 0.87 (95% CI 0.60, 1.26). In subgroup analysis by type of provider, neither nurse nor physician clinical impression had statistically significant positive or negative likelihood ratios for predicting severe disease in our sample of children.

## Discussion

We initially chose to study the WHO severe dehydration scale and CDC scale because of their widespread international endorsement. The WHO severe dehydration scale has been incorporated into the WHO Integrated Management of Childhood Illness guidelines, endorsed by the World Gastroenterology Association, and recommended by WHO for use by both nurses and physicians practicing at health facilities worldwide[13,18,19]. Versions of the CDC scale, in turn, have been endorsed for use by the American College of Emergency Physicians and the American Academy of Pediatrics in the United States and the National Institute for Health and Clinical Excellence (NICE) in the United Kingdom, as well as in multiple management guidelines for pediatric gastroenteritis[6,20-23]. We chose to study the Clinical Dehydration Scale post-hoc because of its recent popularity in the pediatric and emergency medicine literature, which have now made it the most well-studied clinical dehydration scale[9,15,24-27]. 

Several previous studies conducted in high- and middle-income countries have assessed the accuracy of clinical scales for predicting dehydration in children with diarrhea using the criterion standard of percent weight change with rehydration. Gorelick, et al. assessed the accuracy of a 10-point clinical scale in children with diarrhea presenting to a single pediatric referral hospital in Philadelphia, while Vega, et al. assessed the accuracy of a similar 9-point scale in children presenting to an academic medical center in New York[8,10]. Gorelick, et al. found their 10-point scale to have a sensitivity of 82% and specificity of 90% for predicting severe dehydration in children when assessed by an experienced emergency nurse, while Vega, et al. found their 9-point scale to have a sensitivity of 70% and specificity of 84% for predicting severe dehydration when performed by an emergency physician. Duggan, et al. studied the accuracy of two older clinical scales in a population of children presenting to a pediatric gastroenteritis clinic in Cairo, Egypt. All children were assessed clinically by the three study physicians, who found that the two scales were able to discriminate between children with mild, moderate, and severe dehydration[7]. 

The Clinical Dehydration Scale was initially derived in a population of children presenting with diarrhea to a Canadian pediatric referral hospital and found to be a significant predictor of moderate-severe dehydration in that same population of children[9,27]. Additional studies have found the Clinical Dehydration Scale to be a significant predictor of emergency department length of stay, treatment with intravenous fluids, and hospitalization, but not severity of dehydration[24-26].

To our knowledge, the only prior study to assess the accuracy of a specific clinical dehydration scale against an established criterion standard in a low-income country setting was a pilot for this current research study conducted in the same three hospitals in Rwanda[14,15]. This pilot study did not find the WHO severe dehydration scale or Clinical Dehydration Scale to be accurate predictors of severe dehydration in children, though it had several important limitations including a small sample size; the use of final weight instead of stable weight to assess the percent weight change with rehydration; and the exclusion of children who died prior to discharge, which could bias the results towards the null by excluding those children most likely to have high scores on the clinical scales.

Our current study builds upon this prior research, assessing the performance of three popular clinical scales against the criterion standard of severe disease in a low-income country setting. As clinical assessments for children in our study were performed by physicians and nurses without advanced training, the results are likely generalizable to many other resource-limited settings. We find all three scales to be moderate predictors of severe disease in children with diarrhea, with statistically significant AUCs ranging from 0.72 to 0.80. Sub-group analysis, however, suggests that only the Clinical Dehydration Scale remains a statistically significant predictor of severe disease when used by general practice nurses or when used in the assessment of infants. This may be because the signs that make up the Clinical Dehydration Scale are easier to assess in children overall, and especially in children under twelve months, than the signs included in the CDC scale and WHO severe dehydration scale. While there are many similarities between the Clinical Dehydration Scale and the WHO severe dehydration scale in particular, there are still some important differences. Differentiating between slow skin pinch and very slow skin pinch, for instance, may be especially challenging for less-skilled practitioners. 

Interestingly, overall clinical impression was not a statistically significant predictor of severe disease in our study. While clinicians may feel that they can intuitively identify which children with diarrhea are severely dehydrated and which ones are not, this study finds that general practice physicians and nurses are likely to both over and under-diagnose severe dehydration based on their overall clinical impression, suggesting an important role for the use of standardized clinical scales when assessing children with diarrhea.

### Limitations

In our study, we utilize percent weight change with rehydration as our criterion standard for severity of dehydration, as has been the standard in the literature for the past several decades[4,7-10,27]. However, we were only able to assess the severity of dehydration in the 76% of patients who achieved a stable weight prior to discharge. Six percent of children continued gaining weight until discharge, as is common among severely malnourished children with wasting (marasmus) who receive protein-energy supplementation in the hospital. By comparison, 13% of patients lost weight during their hospital stay, as is common among severely malnourished children with edema (kwashiorkor) who generally begin to diurese excess extravascular fluid when treated with protein-energy supplementation in the hospital. Including these children in our analysis would have biased our findings, since their percent weight gain would largely be a function of treatment for their severe malnutrition as opposed to the severity of their dehydration due to diarrhea. As noted in Table 2, the children who did not achieve a stable weight prior to discharge were more likely to have severe malnutrition than the children who did achieve a stable weight, so the accuracy of the three clinical scales found in this study cannot be generalized to children with severe malnutrition. 

The proportion of children with severe disease in our study was several times higher than the expected 2% incidence in children with diarrhea reported in the recent global health literature[1]. This is likely because our study only included patients admitted to the hospital, and as such represents a more acutely ill population of children with diarrhea. However, our study still enrolled children representing the full spectrum of diarrhea severity, from no dehydration to some dehydration to severe dehydration and death. Moreover, measuring serial weights to determine the percent weight change with rehydration in outpatients in our study setting was infeasible, as most children lived in distant rural villages several hours away from our study hospitals and could not be expected to return to the hospital for daily weight checks in order to calculate their percent weight change with rehydration. This same limitation is likely to be present in any study of dehydration in children conducted in a rural, low-income country setting. In addition, we only enrolled children between 7:00am and 5:00pm on weekdays, based on the availability of study staff. It is likely that children admitted with diarrhea at night and on weekends would have had even higher proportions of severe disease than those enrolled in our study (Figure 1).

Our study was powered to detect a difference of at least 0.2 in the area under our ROC curves based on the performance of the best and worst ROC curves in our pilot study[14,15]. Since all three scales had similar AUCs in our overall study population, we were not able to demonstrate that any one scale performed statistically better than any other. While the scales had very different AUCs in two sub-populations (children under 12 months and those assessed by nurses), our study was not powered to detect differences between scales in these sub-populations. Since we did not directly compare the three clinical scales to each other in this study, we have not adjusted our analysis for multiple comparisons. 

We chose to include all children with acute diarrhea in our study, regardless of the final discharge diagnosis. While nearly two-thirds of patients enrolled in our study did eventually receive a diagnosis of gastroenteritis, this diagnosis was not available to our study staff on patient arrival to use as an inclusion criterion. Moreover, gastroenteritis itself can be a relatively subjective diagnosis, especially in settings with limited diagnostic resources. Finally, we chose to use a composite outcome of severe dehydration or death instead of severe dehydration alone in order to avoid biasing the performance of the three clinical scales by excluding the sickest children presenting with diarrhea who are most likely to die prior to being fully rehydrated to a stable weight. The use of the term severe disease to refer to children with severe dehydration or death from diarrhea is also consistent with current nomenclature in the global child health literature[1].

## Conclusion

This is the first study to affirmatively demonstrate that clinical scales can be used to predict severe disease in children with diarrhea in a low-income country setting. The WHO severe dehydration scale, CDC scale, and Clinical Dehydration Scale were all moderate predictors of severe disease in children with diarrhea. Only the Clinical Dehydration Scale was a statistically significant predictor of severe disease when used to assess infants or when used by nurses. However, it remains possible that a new clinical prediction rule specifically derived in a low-income country population of children with diarrhea might perform even better in this setting than the Clinical Dehydration Scale, which was derived in a high-income country. Future research should focus specifically on developing or validating clinical prediction rules for severe disease that can be used by general practice nurses and other less skilled providers in resource-limited settings to accurately evaluate children of all ages with diarrhea.
